# Nasotracheal Intubation After Transsphenoidal Surgery: A Case Report

**DOI:** 10.7759/cureus.24171

**Published:** 2022-04-15

**Authors:** Kanta Kido, Takumi Sato, Hitoshi Miyashita

**Affiliations:** 1 Department of Dental Anesthesiology, Kanagawa Dental University, Yokosuka, JPN; 2 Department of Oral and Maxillofacial Surgery, School of Dentistry, Matsumoto Dental University, Matsumoto, JPN; 3 Division of Oral and Maxillofacial Surgery, Department of Disease Management Dentistry, Graduate School of Dentistry, Tohoku University, Sendai, JPN

**Keywords:** orthognathic surgery, intracranial penetration, transsphenoidal surgery, a balloon catheter, nasotracheal intubation

## Abstract

Nasotracheal intubation is generally used in maxillofacial and oral surgeries under general anesthesia. However, nasal intubation may cause various complications including epistaxis, retropharyngeal dissection, and intracranial penetration of the nasotracheal tube, which occurs in patients with basal skull defects or fractures. Therefore, nasotracheal intubation is usually contraindicated in such patients. Herein, we describe an alternative technique using a balloon catheter in nasotracheal intubation to avoid surgical airway management in a patient with a history of transsphenoidal surgery. The use of a balloon catheter may be a simple and safe method of nasotracheal intubation in patients with basal skull defects.

## Introduction

Nasotracheal intubation is commonly implemented in maxillofacial and oral surgeries performed under general anesthesia. In comparison with oral intubation, however, nasal intubation may cause various complications, such as epistaxis, turbinectomy, local infection, bacteremia, and retropharyngeal dissection [1-3]. Among them, the most feared complication is intracranial penetration of a nasotracheal tube, which can occur in patients with basal skull defects or traumatic fractures of the face and basal skull [4-7]. Therefore, nasotracheal intubation is traditionally contraindicated in such patients [3,6,8,9].

However, if these patients omit to undergo maxillofacial surgery that requires interdental occlusion, for the adjustment and fixation of maxillary fractures or orthognathic surgery, the standard orotracheal intubation may be unsuitable. Therefore, in these cases, anesthesiologists might consider two options for airway management during general anesthesia: the surgical approach, including elective tracheostomy and submental intubation [8,10], and fiberoptic nasotracheal intubation [11]. Although tracheostomy is traditionally used and well established for airway management, the surgical procedure is associated with several complications, including hemorrhage, recurrent laryngeal nerve damage, pneumothorax, tracheal stenosis, and undesirable scarring [10,12]. Submental intubation can also cause hemorrhage, infection, orocutaneous fistula, and injury to the sublingual or lingual nerves [8,12]. Fiberoptic nasotracheal intubation is a standard technique for the management of patients with problematic airways [13]; however, the failure rate of fiberoptic intubation when performed by skillful and experienced anesthesiologists is still 4-5% [14], which is caused by anatomical abnormalities, bleeding, and secretions in the oral and pharyngeal cavities [15,16]. Thus, alternative options for nasotracheal intubation in patients with basal skull defects are needed.

Acromegaly is a rare endocrine disorder that results from the hypersecretion of growth hormone (GH) and insulin-like growth factor 1 (IGF-1) from tumorous pituitary somatotroph cells [17,18]. Chronic hypersecretion of these hormones leads to comorbidities, including arthritis, glucose intolerance, facial changes, mandibular prognathism (protruding lower jaw), and jaw malocclusion. Thus, endoscopic transsphenoidal surgery is performed through the nose to remove pituitary adenomas, which requires the removal of the sellar bone covering the anterior surface of the cavernous sinus [18].

Herein, we report the use of balloon-guided nasotracheal intubation as an alternative technique during orthognathic surgery in a patient with acromegaly and a history of transsphenoidal surgery. Written informed consent was obtained from the patient for the publication of this case report.

## Case presentation

**Figure 1:**
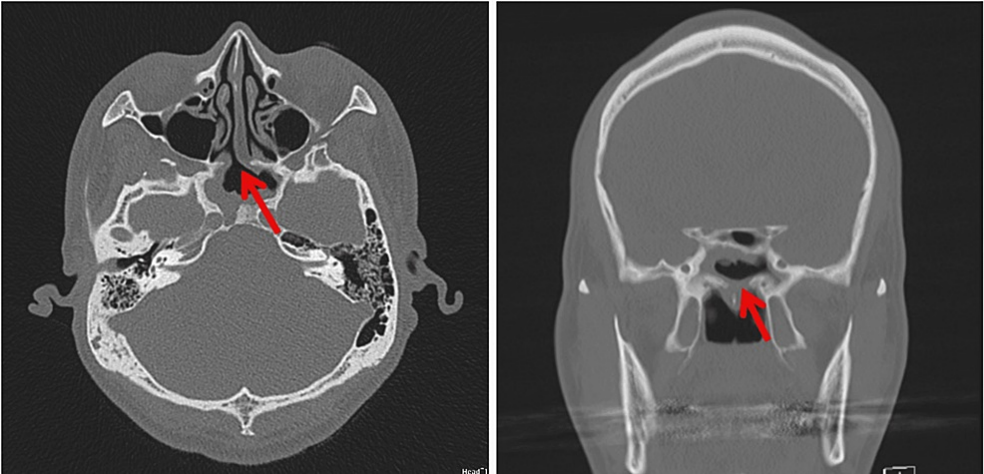
Radiographic examination of the skull base before orthognathic surgery. Left panel: sagittal CT scan demonstrating a bony defect in the sella turcica (red arrow). Right panel: coronal CT scan demonstrating the same bony defect (red arrow).

The patient was a 44-year-old man (height, 170.0 cm; weight, 82.0 kg; body mass index {BMI}, 28.4 kg/m^2^) with a diagnosis of skeletal mandibular prognathism who was scheduled for bilateral sagittal split ramus osteotomies. He was diagnosed with a growth hormone-secreting pituitary adenoma and underwent transsphenoidal surgery 12 years previously. Preoperative computed tomography (CT) of the skull base revealed a bone defect in the sella turcica near the anterior surface of the cavernous sinus (Figure 1), which may lead to serious complications related to intracranial penetration during nasotracheal intubation. Therefore, for airway management during surgery, we attempted an alternative intubation technique using a balloon catheter before fiberoptic intubation or a surgical approach, including elective tracheostomy and submental intubation. 

**Figure 2:**
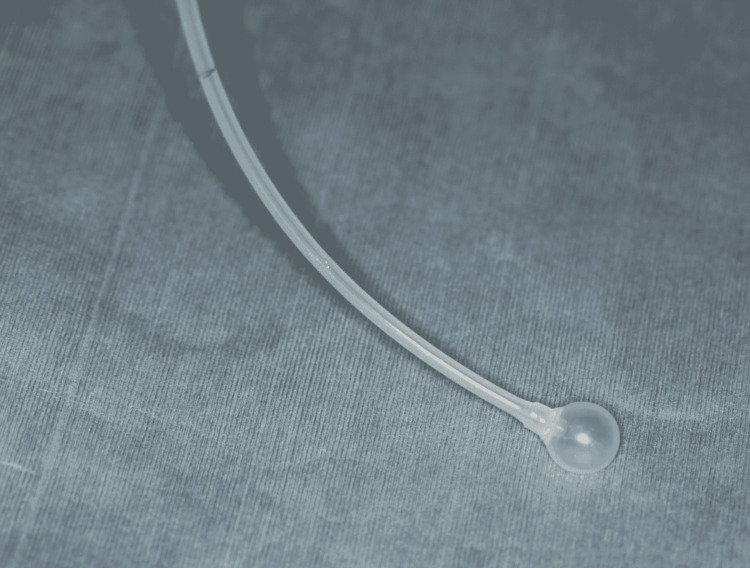
A balloon catheter produced for prevention of epistaxis following balloon inflation Posterior Nasal Cavity Balloon Type (BTM, KOKEN CO. LTD. Tokyo, Japan)

In the operating room, standard Japanese Society of Anesthesiologists (JSA) monitors were used, consisting of electrocardiography, pulse oximeter, noninvasive blood pressure device, bispectral index (BIS) monitor, and body temperature monitor. The end-tidal carbon dioxide and inspired oxygen concentrations were also monitored. After a bolus administration of fentanyl 100 µg and a 3-minute infusion of remifentanil (0.3 µg/kg/min), anesthesia was induced with propofol (initial effect-site concentration 5 µg/mL) administered via a syringe pump (TE-371, Terumo, Inc., Tokyo, Japan) with a built-in target-controlled infusion system (Diprifusor; AstraZeneca, London, UK). Rocuronium (50 mg) was administered for the muscle relaxation required for endotracheal intubation. First, orotracheal intubation was performed using standard direct laryngoscopy with a reinforced 7.5-mm endotracheal tube (Mallinckrodt reinforced tracheal tube; Covidien, Athlone, Ireland). A balloon catheter, which was produced to prevent epistaxis by inflating the balloon (posterior nasal cavity balloon type BTM, KOKEN CO. LTD. Tokyo, Japan), was used to guide nasotracheal intubation (Figure 2).

**Figure 3:**
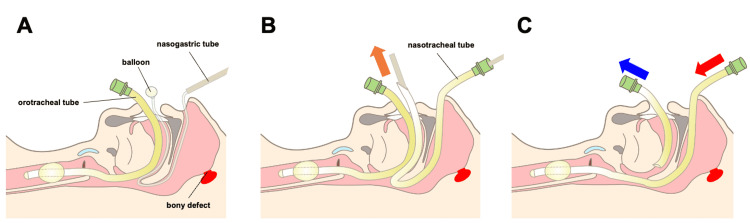
Balloon catheter-guided nasotracheal intubation procedure A. After oral intubation, a lubricated balloon catheter was advanced into the nostril and inflated before reaching the posterior wall of the pharynx; the catheter was then connected to a 16-Fr nasogastric tube. B. A reinforced tube was railroaded over the nasogastric tube from the nostril to out of the mouth. C. An oral endotracheal tube was extubated using a direct laryngoscope and the nasotracheal tube was intubated using a Magill forceps

In the patient, the distance from the external naris to the choana on the preoperative lateral skull radiograph was 56 mm. Thus, the lubricated balloon catheter was advanced 50 mm into the nostril and then inflated with 5 mL air before reaching the posterior wall of the pharynx to avoid intracranial penetration. Next, the catheter was connected to a 16 Fr nasogastric tube and advanced with twisting and vibrating until visualized in the posterior pharynx under direct vision laryngoscopy; the balloon catheter was then pulled out with the aid of a Magill forceps through the patient’s mouth (Figure 3A), and a 7.0-mm reinforced tube was railroaded over the nasogastric tube from the nostril to out of the mouth (Figure 3B). The balloon catheter and nasogastric tube were then removed, and the endotracheal tube was gently placed in the pharynx. Finally, the oral endotracheal tube was extubated using a direct laryngoscope, and the nasotracheal tube was intubated using Magill forceps (Figure 3C). Nasotracheal intubation was confirmed without the tube touching the posterior wall of the pharynx and any complications.

The surgical procedure was completed in 121 min. The patient emerged from anesthesia approximately 15 min after discontinuation of sevoflurane and was extubated easily without any complications. The patient’s subsequent hospital course and recovery were uneventful.

## Discussion

Herein, we describe an alternative technique using a balloon catheter for nasotracheal intubation in a patient with a history of transsphenoidal surgery. Although nasotracheal intubation is believed to be traditionally contraindicated in patients with basal skull defects, this new technique is noninvasive, simple, and safe when compared with tracheostomy or fiberoptic intubation.

The indications for nasotracheal intubation need to be considered along with surrogate techniques, in terms of both their risks and benefits. Orthognathic surgery requires interdental occlusion to adjust and fix the maxillomandibular occlusal state. Therefore, the oral route for tracheal intubation may be unsuitable in such surgeries. In this case, we first considered surgical airway management during surgery to avoid intracranial penetration of a nasotracheal tube attributed to basal skull defects after transsphenoidal surgery. However, the surgical procedure, including tracheostomy and a submental approach, may have potential complications, including bleeding, infection, and hypertrophic scarring [12], which can be relatively notable in patients who undergo elective orthognathic surgery. Fiberoptic nasotracheal intubation was also considered; however, anatomical abnormalities, bleeding, and secretion in the oral and pharyngeal cavity can cause the failure of intubation or accidental intracranial placement of the fiberoptic scope itself [16,19]. Therefore, we devised a simple and safe technique using a balloon catheter as an alternative to fiberoptic intubation.

Various methods, including the use of thermo-softened tubes, guidance with suction catheters, or gum elastic bougies for endotracheal tubes, have been reported to avoid tissue damage during nasotracheal intubation [20]. However, these methods may cause damage to the retropharyngeal submucosa. The balloon catheter used in this case was utilized for the prevention and arrest of epistaxis. By inflating the balloon before reaching the retropharyngeal wall, the catheter could noninvasively pass through the oral cavity without any complications, including retropharyngeal dissection or intracranial penetration. If the balloon is difficult to pass through the retropharyngeal-oral area, the catheter should be gently twisted and vibrated for visualization. If all other approaches fail, fiberoptic nasotracheal intubation should be considered.

## Conclusions

We described a new alternative technique using a balloon catheter for nasotracheal intubation in a patient with a history of transsphenoidal surgery. The use of a balloon catheter may be a simple and safer method for nasotracheal intubation in patients with basal skull defects. This method may be considered before a surgical airway or fiberoptic nasotracheal intubation is performed. 
